# A new species of *Hyalella* (Crustacea, Amphipoda, Dogielinotidae) from the Atlantic Forest of Misiones, Argentina

**DOI:** 10.3897/zookeys.481.9037

**Published:** 2015-02-04

**Authors:** María Florencia Colla, Inés Irma César

**Affiliations:** 1 División Zoología Invertebrados, Facultad de Ciencias Naturales y Museo - FCNyM, Universidad Nacional de La Plata - UNLP, Av. Paseo del Bosque, s/n°, 1900, La Plata, Bs. As., Argentina; 2 Member of the Consejo Nacional de Investigaciones Científicas y Técnicas (CONICET); 3 Member of the Comisión de Investigaciones Científicas de la provincia de Buenos Aires (CIC)

**Keywords:** *Hyalella*, taxonomy, freshwater amphipods, novel-species description, Argentina

## Abstract

The freshwater genus *Hyalella* Smith, 1874 has a distribution restricted to the Western Hemisphere with most species being found in South America. In this report we describe a new species of *Hyalella* from the Atlantic Forest of the Misiones province, Argentina.

## Introduction

The genus *Hyalella* includes approximately 70 valid species distributed in only the Americas (Baldinger 2004, WoRMS 2014). The hyalellids inhabit different freshwater environments, associated with either the bottom sediments (benthic fauna) or the aquatic vegetation (Poi de Neiff 1992), where these amphipods constitute a fundamental link in the transfer of matter and energy in those ecosystems (Casset et al. 2001, González et al. 2006, Castiglioni and Bond-Buckup 2008).

Currently, nine species of *Hyalella* have been recorded for Argentina: *Hyalella curvispina* Shoemaker, 1942, *Hyalella fossamancinii* Cavalieri, 1959, *Hyalella pampeana* Cavalieri, 1968, *Hyalella neonoma* Stock & Platvoet, 1991, *Hyalella falklandensis* Bousfield, 1996, *Hyalella rionegrina* Grosso & Peralta, 1999, *Hyalella araucana* Grosso & Peralta, 1999, *Hyalella kochi* González & Watling, 2001, and *Hyalella bonariensis* Bond-Buckup, Araujo & Santos, 2008 (Santos et al. 2008, De los Ríos-Escalante et al. 2012). Although studies on the genus have increased in recent years, essential aspects of the taxonomy and ecology of *Hyalella* in Argentina still remain poorly known.

The Atlantic Forest of South America – a species-rich and ecologically highly complex system – is considered one of the biodiversity “hot spots” of the world (Myers et al. 2000). In Argentina, the Atlantic Forest includes the province of Misiones, where part of the remaining forest biome is partially protected by the Yabotí Biosphere Reserve.

The aim of this work was to describe a new species of freshwater amphipod of the genus *Hyalella* from the Atlantic Forest in Misiones, Argentina.

## Materials and methods

**Figure 1.**
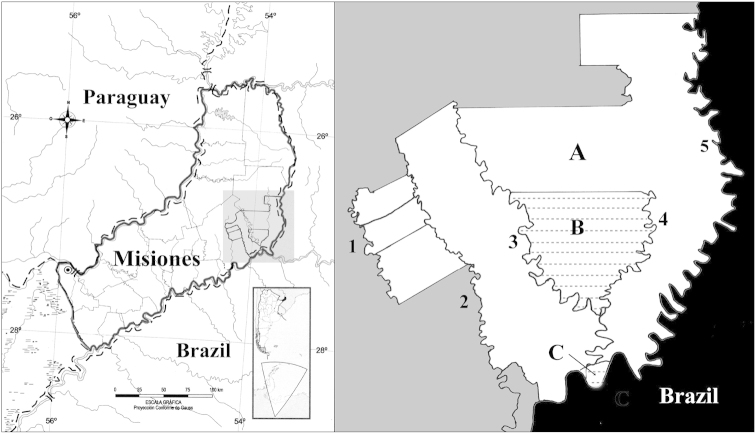
Map of the study area. Left panel: Location of the Province of Misiones, Argentina and the Yabotí Biosphere Reserve in the eastern-central portion. Right panel: **A** Yabotí Biosphere Reserve (entire white area) **B** Esmeralda Provincial Park (textured area) **C** Moconá Provincial Park (textured area). **1** Soberbio stream. **2** Paraíso stream. **3** Yabotí stream. **4** Yabotí Miní stream. **5** Pepirí Guazú River.

The Yabotí Biosphere Reserve is located in the eastern central region of the Misiones province (Fig. 1). The climate is hot and humid without dry season, with an annual mean precipitation of 2000 mm and an annual mean temperature of 21 °C (Cabrera 1971).

Amphipods were collected by hand from the epilithic vegetation (bryophytes) growing on the rocks of the waterfall Salto Isipós, near Paraíso stream (27°13.19'S; 54°02.73'W).

In the laboratory, the cephalothorax length (CL) and total length (TL) of 30 specimens (15 males and 15 females) was measured under a stereoscopic microscope with a milimetric scale (LEICA EZ4). The anatomical pieces were placed in semipermanent slides and the drawings realized by means of a drawing tube mounted on a microscope ocular (LEICA DMLS). The terminology used for the setae of the appendices follows Zimmer et al. (2009). Morphological description is generalized from 10 individuals dissected (5 males and 5 females). We have mentioned the variations when appropiate.

Type material is deposited on Colección de Carcinología, División Zoología Invertebrados (DZI), Facultad de Ciencias Naturales y Museo (FCNyM), Universidad Nacional de La Plata (UNLP), Argentina.

## Taxonomy

### Family Dogielinotidae Gurjanova, 1953 Subfamily Hyalellinae Bulycheva, 1957 Genus *Hyalella* Smith, 1874

#### Hyalella misionensis sp. n.

Taxon classificationAnimaliaAmphipodaDogielinotidae

http://zoobank.org/02941807-666D-4A05-8A3E-80DACD6F0FBA

##### Type material.

Holotype male, Argentina, Province of Misiones, Yabotí Biosphere Reserve, San Pedro and Guaraní Departments, Salto Isipós (27°13.19'S; 54°02.73'W) (MLP 26978), October, 19, 2011. César, I. I. and Martín, S. M., collectors.

##### Paratypes.

15 males, 15 females, 7 ovigerous females, and 70 juveniles (MLP 26979), same data as holotype.

##### Type locality.

Argentina, Province of Misiones, Yabotí Biosphere Reserve, San Pedro and Guaraní Departments, Salto Isipós (27°13.19'S; 54°02.73'W).

##### Diagnosis.

Body surface smooth. Coxa 4 excavated posteriorly. Eyes pigmented. Antenna 1 shorter than antenna 2. Antenna 2 less than half the body length.Maxilla 1 palp short, reaching to less than half the distance between base of palp and tip of setae on outer plate; inner plate slender, with two strong, pappose apical setae. Maxilla 2 with two strong pappose setae on inner margin. Gnathopod 1 propodus length less than twice maximum width, hammer-shaped, inner face with six to nine serrate setae, comb scales on distoposterior border. Gnathopod 2 propodus ovate, palm shorter than posterior margin, distal margin of palm irregular. Pereiopods 3 and 4 merus and carpus posterior margin with three hind marginal clusters of short setae; propodus posterior margin with five groups of setae. Uropod 3, peduncle slender (rectangular), wider than ramus, with five strong distal setae, basal width more than twice apex of ramus. Telson as long as wide, entire, apically rounded, bearing two long simple setae symmetrically distributed on distal margin, and three small submarginal setae close to each main setae. Sternal gills present on segments 2 to 7.

##### Description of male

**Figure 2.**
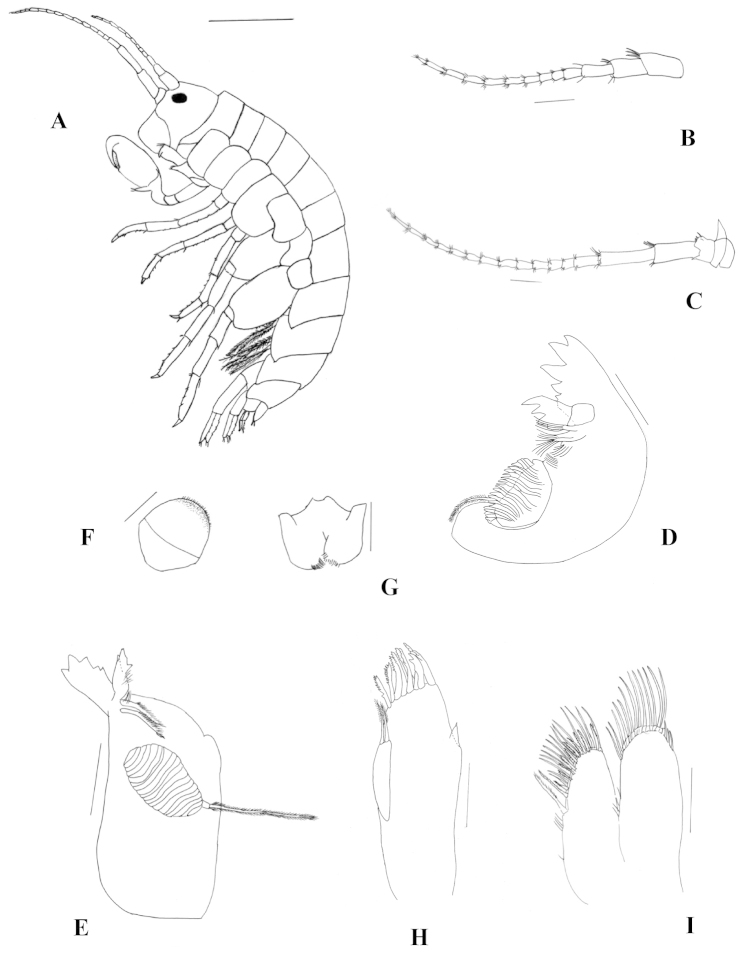
*Hyalella misionensis* sp. n., male. **A** holotype and habitus **B** antenna 1. **C** antenna 2 **D** left mandible **E** right mandible. **F** upper lip **G** lower lip **H** maxilla 1. **I** maxilla 2. Scale bar equals 1 mm for **A**; 200 µm for **B**, **C**, **F**, and **G**; 100 µm for **D**, **E**, **H**, and **I.**

**Figure 3.**
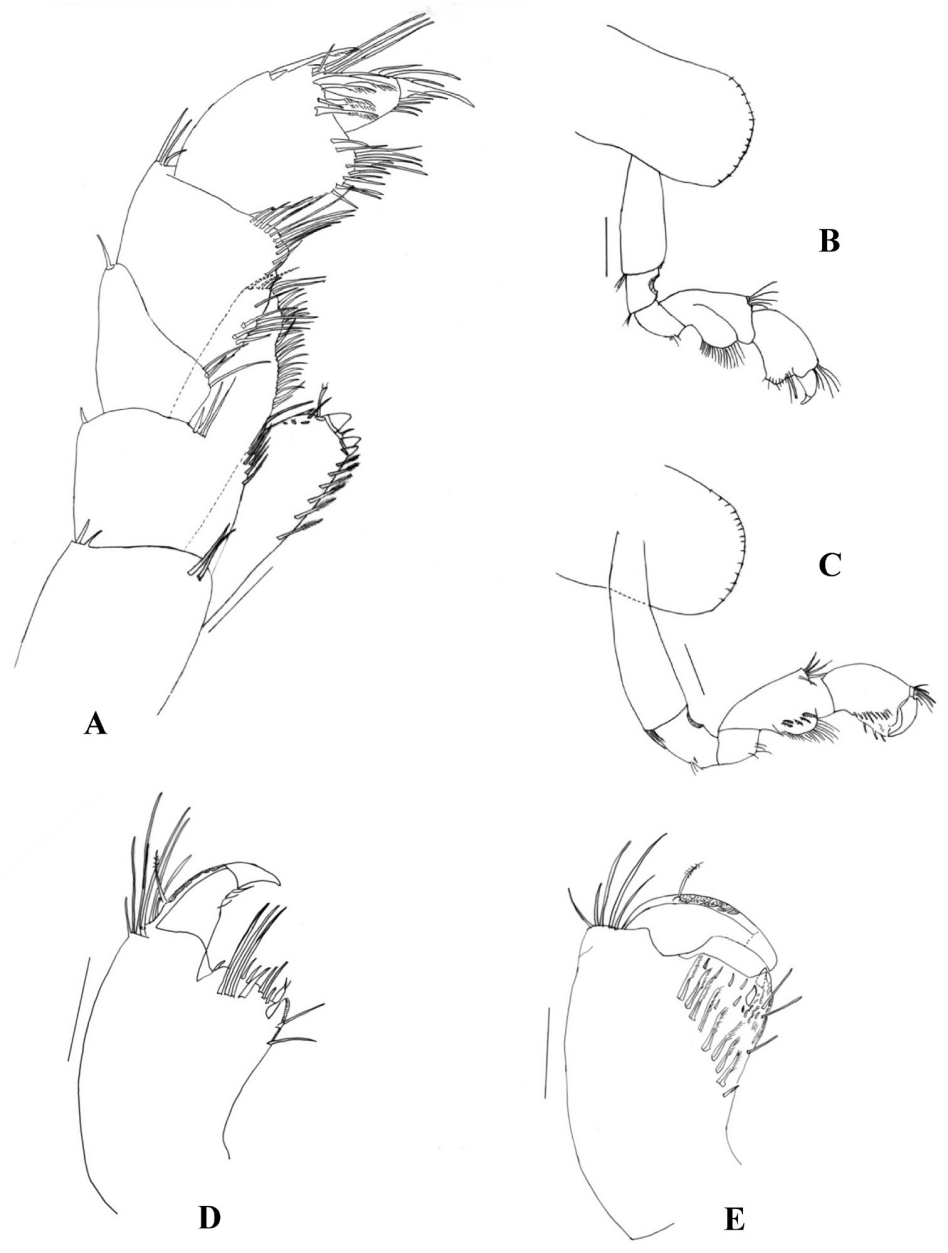
*Hyalella misionensis* sp. n., male. **A** maxilliped **B** dorsal view of gnathopod 1 **C** ventral view of gnathopod 1 **D** dorsal view of propodus of gnathopod 1 **E** ventral view of propodus of gnathopod 1. Scale bar equals 100 µm for **A**, **D**, and **E**; 200 µm for **B** and **C.**

**Figure 4.**
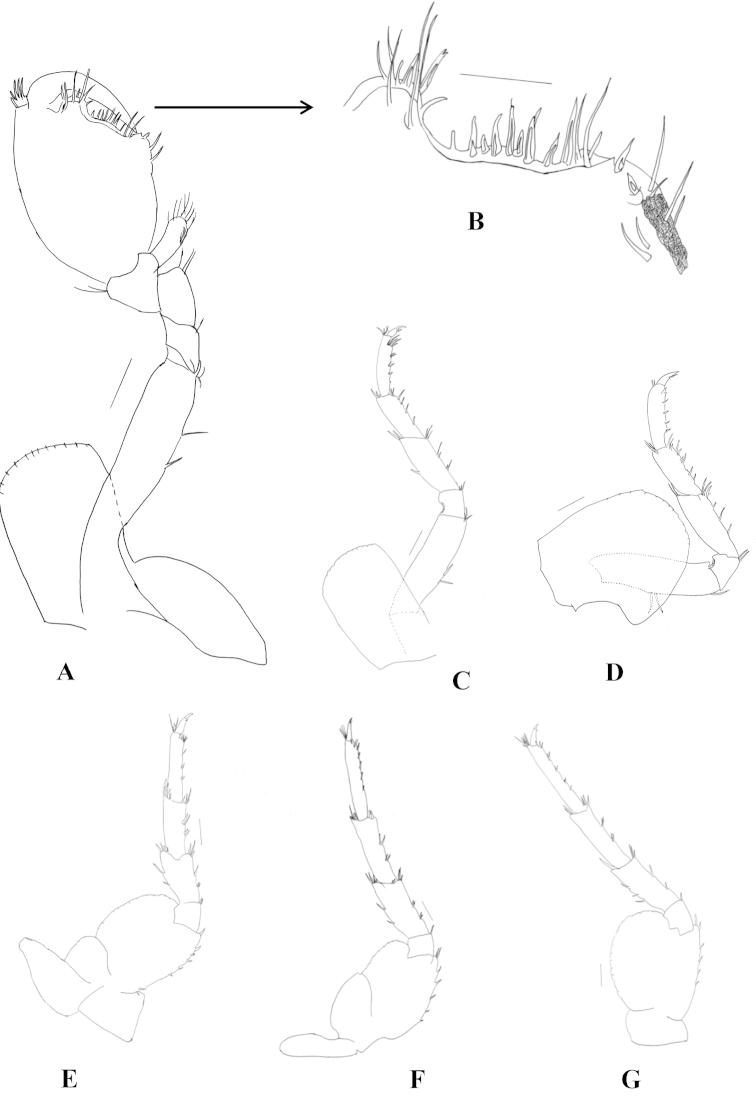
*Hyalella misionensis* sp. n., male. **A** gnathopod 2 **B** detail of the palm of gnathopod 2 **C** pereiopod 3 **D** pereiopod 4 **E** pereiopod 5 **F** pereiopod 6 **G** pereiopod 7. Scale bar equals 200 µm for **A**, **C**, **D**, **E**, **F**, and **G**; 100 µm for **B.**

**Figure 5.**
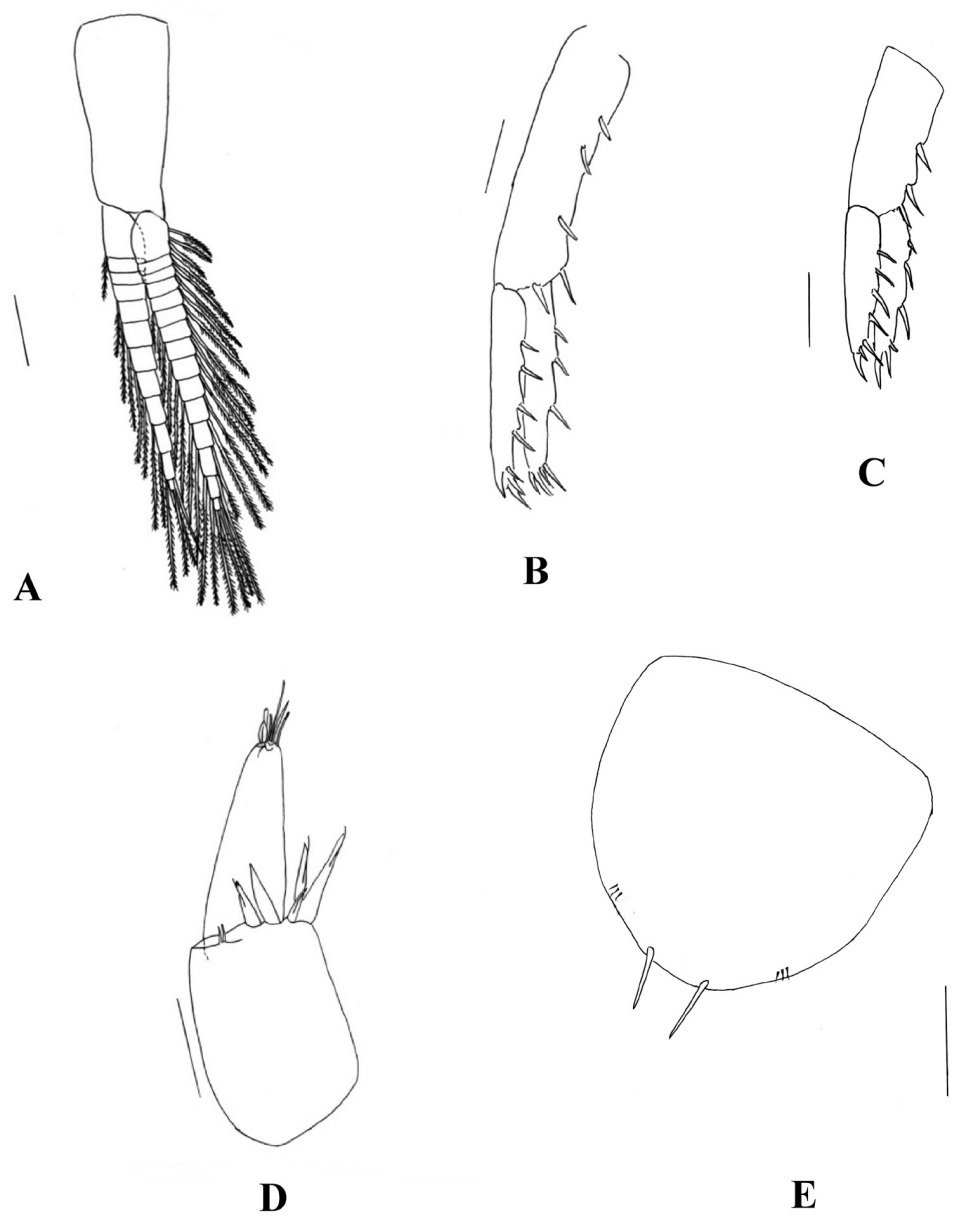
*Hyalella misionensis* sp. n., male. **A** pleopod **B** uropod 1 **C** uropod 2 **D** uropod 3 **E** telson. Not all setae are represented in the figures. Scale bar equals 200 µm for **A**, **B**, and **C**; 100 µm for **D** and **E.**

(Figs 2 to 5). Mean body length: 5.9 ± 1.09 mm; mean cephalothorax length: 0.61 ± 0.08 mm (n = 15). Body surface smooth. Epimeral plate 1, 2, and 3 acuminate. Coxae 1 to 4 subequal in size and shape, slightly overlapping. Acumination in coxae absent. Coxa 1 similar to 2 and 3. Coxa 3 narrower than 4. Coxa 4 as wide as deep, excavated posteriorly. Coxa 5 posterior lobe deeper than anterior lobe. Coxa 6 posterior lobe deeper than anterior lobe, anterior lobe small.

Head typically gammaridean, as long as the first two thoracic segments, rostrum absent. Eyes pigmented, medium, rounded, located between insertion of antenna 1 and antenna 2 (Fig. 2A).

Antenna 1 (Fig. 2B) less than half the body length, shorter than antenna 2 but longer than peduncle of antenna 2; peduncle as long as head; article 3 shorter than article 1 and article 2 in length; flagellum of 10–11 articles, longer than peduncle; aesthetascs on flagellum, present on articles 4 (2 aesthetascs), 5 (2), 6 (3), 7 (1), and 8 (1).

Antenna 2 (Fig. 2C) less than half of the body length, peduncle longer than head, article 4 shorter than article 5, setal groups on articles 4 and 5 scarce, flagellum with 13–14 articles and longer than peduncle.

Mandible without palp; incisor toothed; left lacinia mobilis with five teeth; setal row on left mandible with three main pappose setae plus accessory setae (Fig. 2D), right mandible with two main pappose setae plus accessory setae; molar large, cylindrical, and triturative; accessory seta present (Fig. 2E).

Upper-lip ventral margin round (Fig. 2F).

Lower-lip outer lobes rounded without notches or excavations, mandibular projection of outer lobes truncated (Fig. 2G).

Maxilla 1 (Fig. 2H) palp short, uniarticulate, reaching to less than half the distance between base of palp and tip of setae on outer plate, distally pointed; inner plate slender, smaller than outer plate, with two strong, pappose apical setae; outer plate with nine stout and serrate setae.

Maxilla 2 (Fig. 2I) inner plate subequal in length and width to outer plate, with two strong pappose setae on mid-inner margin; outer and inner plates with abundant setules.

Maxilliped (Fig. 3A) inner plates apically truncated, with three connate setae and pappose setae apically and medially; outer plates larger than inner plates, apically truncated, apical, medial, and facial setae simple. Palp of four articles: article 2 wider than long, medial margin with long simple setae; article 3 outer distal face (at the base of article 4) with several long simple setae, inner distal face with long plumose setae, inner distal margin with long setae, outer margin with one or two strong and long plumose setae; dactylus unguiform, shorter than article 3, distal setae simple and shorter than nail, inner margin with setae, distal nail present.

Gnathopod 1 (Fig. 3B and C) subchelate; carpus longer than wide, longer and wider than propodus, with strong and wide posterior lobe, and forming a scoop-like structure, open to the inside, inner face with five serrate setae; propodus (Fig. 3D) length less than two times maximum width, hammer-shaped, with no setae on anterior border, with three simple setae on posterior border; inner face (Fig. 3E) with six to nine serrate setae, several small triangular setae, comb scales on distoposterior border, palm slope transverse, margin convex, posterior distal corner with robust setae, dactylus claw-like with comb scales.

Gnathopod 2 (Fig. 4A) subchelate; basis hind margin with two long setae; merus with less than seven setae on posterior margin, posterodistal margin straight, distal corner rounded; carpus posterior lobe elongated, produced between merus and propodus, distal end of carpal lobe with cuticular denticles and with several serrate setae; propodus ovate, distoposterior border with comb scales, palm (Fig. 4B) shorter than posterior margin, slope oblique, margin irregular, bearing several strong short setae, anterior edge with a wide truncated or rounded process, posterior distal corner with strong setae and with cup for dactylus; dactylus claw-like, as long as palm, with seven short simple setae symmetrically distributed on inner border. Triangular space between propodus and dactylus when dactylus is closed.

Pereiopods 3 to 7 simple. Pereiopods 3 (Fig. 4C) and 4 (Fig. 4D) with merus and carpus posterior margin having three hind marginal clusters of short setae; propodus posterior margin with five groups of setae; dactylus less than half the propodus length. Pereiopods 5 to 7, all similar in structure and successively slightly longer; dactylus less than half the propodus length. Pereiopod 5 (Fig. 4E) longer than pereiopod 4, basis posterior lobe longer than wide, smaller than posterior lobe of pereiopod 7. Pereiopod 6 (Fig. 4F) longer than pereiopod 4, basis posterior lobe longer than wide, larger than posterior lobe of pereiopod 5 and smaller than posterior lobe of pereiopod 7. Pereiopod 7 (Fig. 4G) slightly longer than pereiopod 6, basis posterior lobe longer than wide.

Pleopods (Fig. 5A) not modified; peduncle slender; longest ramus longer than peduncle.

Uropod 1 (Fig. 5B) longer than uropod 2; peduncle longer than rami, with 4-5 dorsal setae; rami subequal; inner ramus with 2–3 dorsal setae and 5 distal setae; male without curved setae on inner side of the ramus; outer ramus with 3–4 dorsal setae and 5 distal setae.

Uropod 2 (Fig. 5C) peduncle as long as rami, with 3 dorsal setae; rami subequal; inner ramus with 3 dorsal setae and 6 distal setae, outer ramus with 4 dorsal and 4 distal setae.

Uropod 3 (Fig. 5D) as long as peduncle of uropod 2; peduncle slender (rectangular), wider than ramus, with 5 strong distal setae of variable length, inner ramus absent; outer ramus uniarticulate, as long as peduncle, basal width more than twice apex of ramus, with 4–5 simple slender apical setae and one connate seta.

Telson (Fig. 5E) as long as wide, entire, apically rounded, bearing two long simple setae symmetrically distributed on distal margin, and three small setae close to each main seta.

Coxal gills sac-like, present on segments 2 to 6. Sternal gills tubular, present on segments 2 to 7.

##### Female

**Figure 6.**
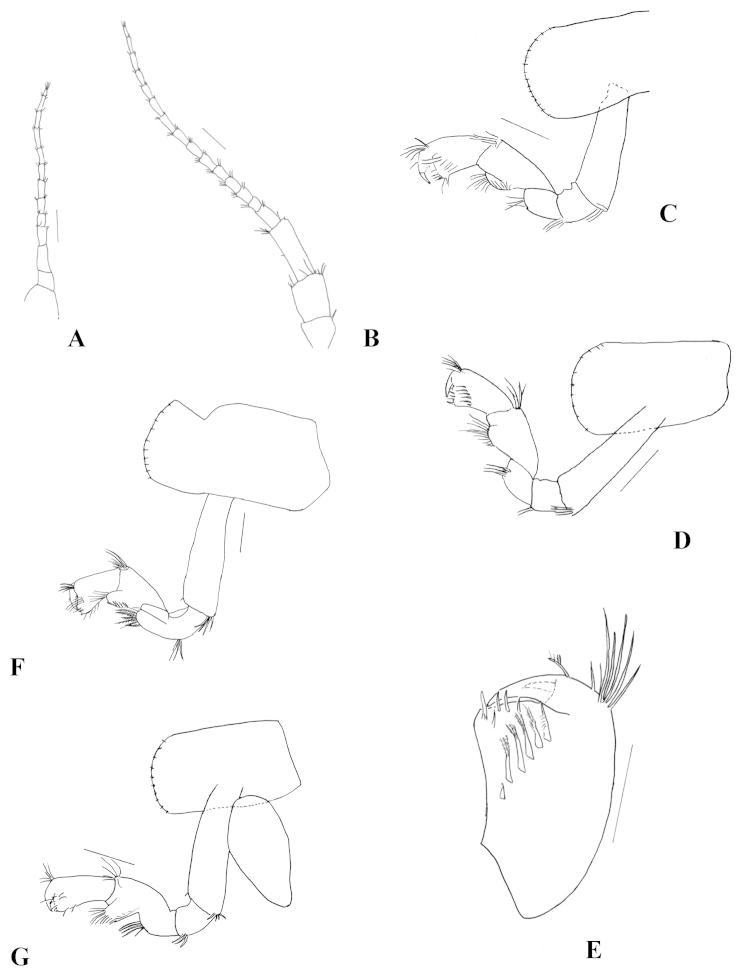
*Hyalella misionensis* sp. n., female. **A** antenna 1 **B** antenna 2 **C** dorsal view of gnathopod 1 **D** ventral view of gnathopod 1 **E** ventral view of propodus of gnathopod 1 **F** dorsal view of gnathopod 2 **G** ventral view of gnathopod 2. Scale bar equals 200 µm for **A**, **B**, **C**, **D**, **F**, and **G**; 100 µm for **E.**

(Fig. 6). Mean total length: 4.52 ± 0.71 mm; mean cephalothorax length: 0.51 ± 0.07 mm (n = 15). Antenna 1 (Fig. 6A) flagellum of 9–10 articles. Antenna 2 (Fig. 6B) similar in length and shape to male, flagellum of 15 articles.

Gnathopod 1 (Fig. 6C and D) similar in size and shape to gnathopod 2. Propodus inner face with six serrate setae (Fig. 6E). Gnathopod 2 (Figs 6F and G) propodus of length less than two times maximum width, normally subchelate, palm transverse. Propodus inner face with two serrate setae.

##### Habitat.

Freshwater, epigean.

##### Distribution.

Argentina, Province of Misiones, Yabotí Biosphere Reserve, San Pedro and Guaraní Departments, Salto Isipós (27°13.19'S; 54°02.73'W).

##### Etymology.

The species name *misionensis* refers to the location where this new member was found, the Province of Misiones in Argentina.

##### Remarks.

*Hyalella misionensis* has some morphological similarities to *Hyalella pampeana* Cavalieri, 1968, a common freshwater amphipod of the Province of Buenos Aires. The principal similarity is the shape of gnathopod 2 in the males, with a triangular space between the propodus and the dactylus in both species; the number of antennal segments (*Hyalella misionensis*, antenna 1: 10–11 articles and antenna 2: 13–14 articles; *Hyalella pampeana*, antenna 1: 11–12 articles and antenna 2: up to 18); and the total length (*Hyalella misionensis*: 5.9 mm, *Hyalella pampeana*: 5 mm). Although, the two species differ in the presence of a curved seta in the inner ramus of uropod 1 in the males of *Hyalella pampeana*; this seta is absent in *Hyalella misionensis*. The width of the propodus of gnathopod 1 is about 3/4 of its length in *Hyalella pampeana*, but about 2/3 of its length in *Hyalella misionensis*. In addition, the inner face of propodus in gnathopod 1 of *Hyalella pampeana* has 5 to 6 pappose setae, but in *Hyalella misionensis* has 6 to 9 serrate setae. The setation of the telson is also different with 2 to 5 setae of variable length in *Hyalella pampeana* and only two simple setae in *Hyalella misionensis*. Comparisons between morphological characters of *Hyalella* species of Argentina and Falkland Islands are presented in Table 1.

**Table 1.** Morphological characters of *Hyalella* species of Argentina and Falkland Islands. All characters are referred to males, with exception of *Hyalella rionegrina*.

Species	Curved setae in inner ramus of uropod 1	Articles of flagellum of antenna 1	Articles of flagellum of antenna 2	Sternal gills	Inner face of propodus of gnathopod 1	Telson
*Hyalella curvispina*	Present	11	13	3–7	5–7 setae	Wider than long, with 3 simple spines.
*Hyalella pampeana*	Present	11–12	Up to 18	2–7	5–6 setae	As wide as long, apically rounded, with 2–5 spines.
*Hyalella falklandensis*	Present	10	12	2–6	5 setae	Wider than long, broadly rounded apex with 4–5 short fine spines.
*Hyalella bonariensis*	Present	9–12	12–15	2–7	5 setae	Quadrangular, with 2–3 cuspidate setae apically.
*Hyalella kochi*	Present	9	11	3–7	7 setae	As wide as long, apically truncated, with two long simple setae.
*Hyalella fossamancinii*	Absent	9–10	9–14	3–7	More than 10 setae	Wider than long, with more than two small or minute setae.
*Hyalella neonoma*	Absent	12	25	3–7	8–10 setae	Wider than long, apex rounded, with 3–4 very small slender spines.
*Hyalella araucana*	Absent	9	12	3–7	10–11 setae	As wide as long, apically rounded, with more than two small or minute setae.
*Hyalella misionensis*	Absent	10–11	13–14	2–7	6–9 setae	As long as wide, apically rounded, with two long simple setae on distal margin.
*Hyalella rionegrina* (female)	?	4	5	2–7	?	Semieliptic, with 4 simple spines.

The study area where *Hyalella misionensis* was found is geographically close to Brazil, where fourteen species of the genus have been reported (Bueno et al. 2013). In Table 2 we compared the main morphological characters of *Hyalella misionensis* with those of the Brazilian *Hyalella* species that were geographically close to where the newly described species was found: *Hyalella castroi* Gonzalez, Bond Buckup & Araujo, 2006, *Hyalella pleoacuta* Gonzalez, Bond Buckup & Araujo, 2006, *Hyalella gracilicornis* Faxon, 1876, *Hyalella longistila* Faxon, 1876 and *Hyalella warmingi* Stebbing, 1899 (Gonzalez and Watling 2003, Gonzalez et al. 2006).

**Table 2.** Main morphological differences between *Hyalella misionensis* and five *Hyalella* species from nearby areas in Brazil.

Species	Body surface	Length of antenna 2	Inner margin of maxila 2	Inner face of propodus of gnathopod 1	Telson	Type locality
*Hyalella misionensis*	Smooth	Less than half the body length	Two pappose setae	6–9 serrate setae	As long as wide, with 2 simple setae	Salto Isipós, Province of Misiones, Argentina
*Hyalella castroi*	Smooth	More than half the body length	One pappose setae	More than 10 serrate setae	Wider than long, with 8 setae	Vale das Trutas, Rio Grande du Sul, Brazil
*Hyalella pleoacuta*	With dorsal flanges on some segments	More than half the body length	Two pappose setae	9 serrate setae	As long as wide, with 2 simple setae	Vale das Trutas, Rio Grande du Sul, Brazil
*Hyalella gracilicornis*	Smooth	More than half the body length	One pappose setae	4 pappose setae	As long as wide with 2 simple setae	Campos, Rio de Janeiro, Brazil
*Hyalella longistila*	Smooth	More than half the body length	One pappose setae	5 pappose setae	Longer than wide, with 2 simple setae	Swamp 3 miles from Campos, Rio de Janeiro, Brazil
*Hyalella warmingi*	Smooth	More than half the body length	One pappose setae	10 pappose setae	Longer than wide, with 2 simple setae	Lagoa Santa, Minas Gerais State, Brazil

## Acknowledgements

Financial support for this work was provided by National Agency for Scientific and Technological Promotion, (Scientific and Technological Research Project, PICT 2042-2008) and by Scientific Research Commission of Buenos Aires province (CIC), Argentina.

We thank Dr. Donald F. Haggerty, a retired career investigator and native English speaker, who edited the final version of the manuscript.

## Supplementary Material

XML Treatment for Hyalella misionensis
